# Penetration of the Scalp by the Bent Fragment of a Pre-installed Titanium Mini-Plate Due to a Minor Head Injury: A Case Report

**DOI:** 10.7759/cureus.78472

**Published:** 2025-02-04

**Authors:** Shoko M Yamada, Shouichi Nakajima, Yoshihiro Hashimoto

**Affiliations:** 1 Neurosurgery, Shizuoka Welfare Hospital, Shizuoka, JPN; 2 Neurosurgery, Teikyo University Mizonokuchi Hospital, Kawasaki, JPN

**Keywords:** bone flap fixation, burr hole, craniotomy, head injury, titanium mini-plate

## Abstract

Titanium mini-plates are commonly used for bone flap fixation in craniotomy and are particularly essential for covering burr holes. Plate exposure through the scalp may occur because of scalp thinning caused by infection or local ischemia, and penetration of the scalp by a titanium mini-plate that had been bent by a minor head injury are rare. A 69-year-old man who had undergone covering of a burr hole in the calvarium by a titanium plate for clipping of a ruptured aneurysm 17 years ago was referred to our clinic to examine metal protruding through the scalp. One year previously, he had hit his head on a wall with no consequence until one week ago, when he noted the protruding metal. Plain skull radiography showed that a portion of the pinwheel-shaped titanium mini-plate had been bent upward and was exposed through the scalp. As the wound was contaminated with sebum, the plate and three screws securing it were removed through a linear skin incision under local anesthesia. The wound healed uneventfully, and the sutures were removed 10 days later. Bone flap fixation requires careful attention. When a five-screw hole pinwheel-shaped titanium mini-plate is used to cover a burr hole, all five holes should be fixed to the skull/bone flap to prevent bending in the future. Alternatively, a wing that is not fixed with a screw should be cut or a rectangular or diamond-shaped plate should be used.

## Introduction

Titanium plates are commonly used for bone fragment fixation in craniotomy and plastic surgery [1-6]. Particularly, titanium mini-plates are essential for covering burr holes [7]. However, it is not uncommon for the implanted titanium plate to be exposed and forced to be removed [3,8,9]. Plate exposure is generally caused by thinning of the scalp, which can result from infection, friction caused by the plates, and skin ischemia at the plate area [3]. Such plate exposure can occur even in proper plate implantation and is difficult to prevent. We present a patient with a history of craniotomy who experienced exposure of a titanium mini-plate through the scalp after a minor head injury. The patient hit his head precisely at the part of the mini-plate that was not fixed to the skull with a screw. The impact caused a portion of the plate to bend and penetrate the scalp. Titanium mini-plates are very thin (0.3-0.5 mm in thickness) and fit firmly to the skull, so it is extremely rare for a titanium mini-plate to be bent vertically by a minor head injury and the bending portion to penetrate the scalp [10]. This situation could have been prevented if all mini-plate holes had been firmly screwed to the skull, and the current case is reported here to emphasize the importance of paying more attention to the titanium mini-plate fixation.

## Case presentation

**Figure 1:**
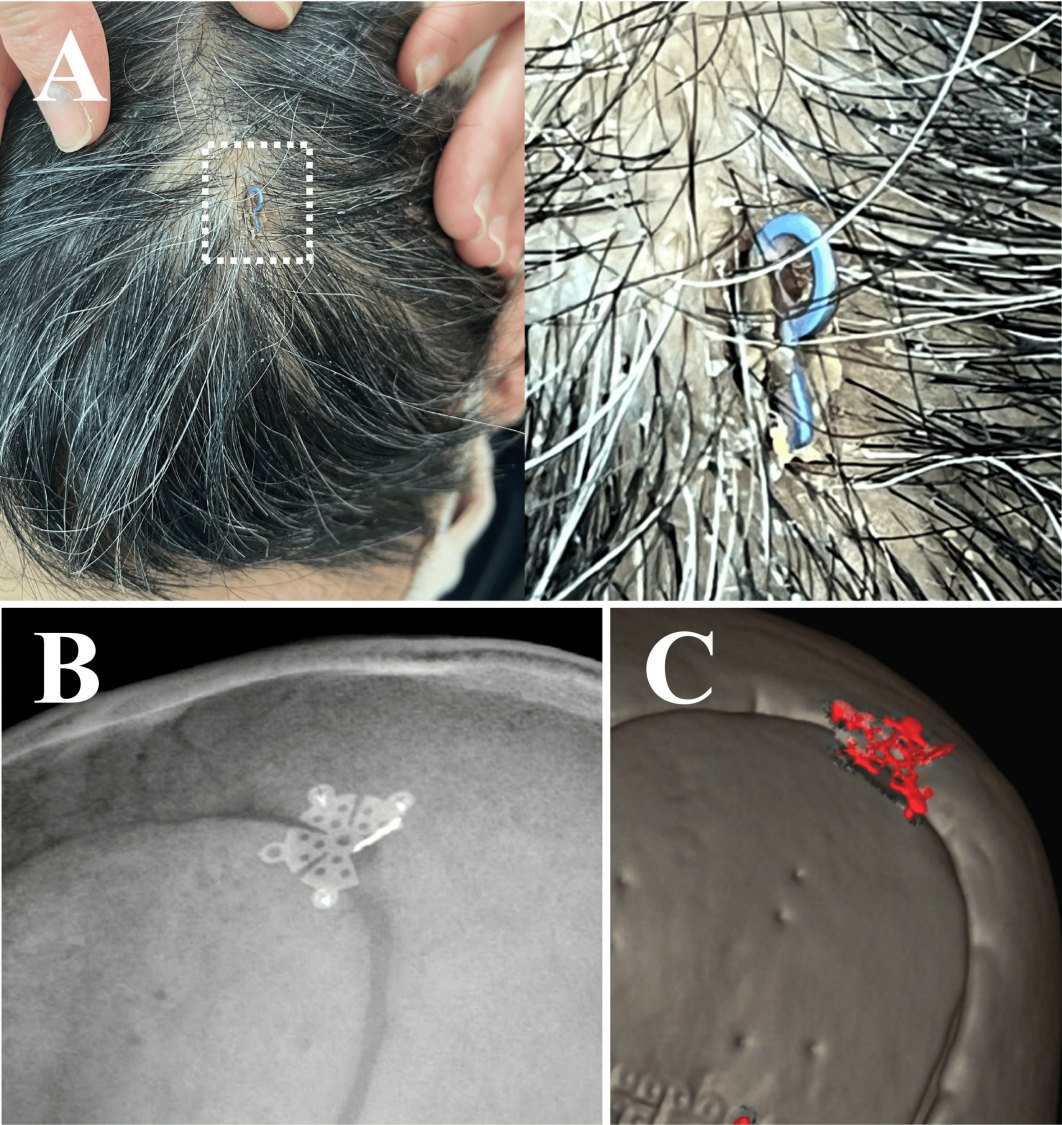
Titanium mini-plate protruding through the scalp A: A light blue metal fragment is seen protruding through the left parietal scalp, showing a screw hole contaminated with sebum. B, C: Plain radiography and three-dimensional computed tomography showed that a pinwheel-shaped, mini-titanium plate covering the burr hole had been fixed using only three screw holes. The unfixed wing was bent upward.

A 69-year-old man underwent craniotomy 17 years ago for a ruptured cerebral aneurysm and recovered to live a normal life. Since no neurological deficits were recognized and no scalp infection was identified in the patient, the postoperative follow-up was completed in three years. He later developed renal failure and had been receiving dialysis 3 times a week for more than 10 years. One year ago, he hit and bruised his head on a wall, with no consequence until one week previously, when he became aware of scalp pain. He was examined by his nephrologist, who found metal exposed through the scalp and referred him to our clinic. On examination, part of the titanium mini-plate was exposed through the scalp, which was covered with filthy sebum (Figure 1A: square dotted line). He was alert without neurological deficits, and his body temperature was within the normal range, as were laboratory indicators of inflammation. Plain skull radiography and three-dimensional computed tomography of the head showed that a pinwheel-shape titanium mini-plate with five screw holes was fixed at the burr-hole portion using three screws and an unscrewed portion of the plate had bent vertically (Figures 1B, 1C).

**Figure 2:**
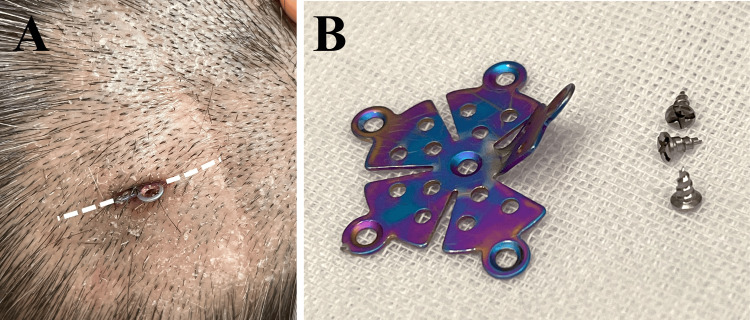
Removal of the plate A: The skin at the plate penetrating area was reddened, suggesting mild inflammation. A 3-cm linear skin incision was made along the direction of the torn skin. In case the wound was too small to identify the screws to be removed, an L-shaped extension incision could have been made using the skin incision from the previous craniotomy. B: The bent plate and three screws were successfully removed through the incision. The deformed shape of the mini-plate was consistent with its shape in imaging studies.

As the risk of infection from the exposed titanium plate to the bone flap was high, plate removal was performed. Under local anesthesia, the plate was removed through a 3 cm linear skin incision that included the protruding plate in its center (Figure 2A). Once the subcutaneous tissue covering the plate had been peeled off, three screws were removed from the skull/bone flap using a screwdriver, and finally, the plate was removed by floating it with a surgical dissector. The removed plate and screws are shown in Figure 2B. The fragile erythematous skin was debrided and the wound was irrigated thoroughly with a gentamicin-containing saline solution. The incision was then closed using a 4-0 nylon suture. The skin incision area healed well without any signs of infection and the sutures were removed 10 days later in our clinic.

## Discussion

**Figure 3:**
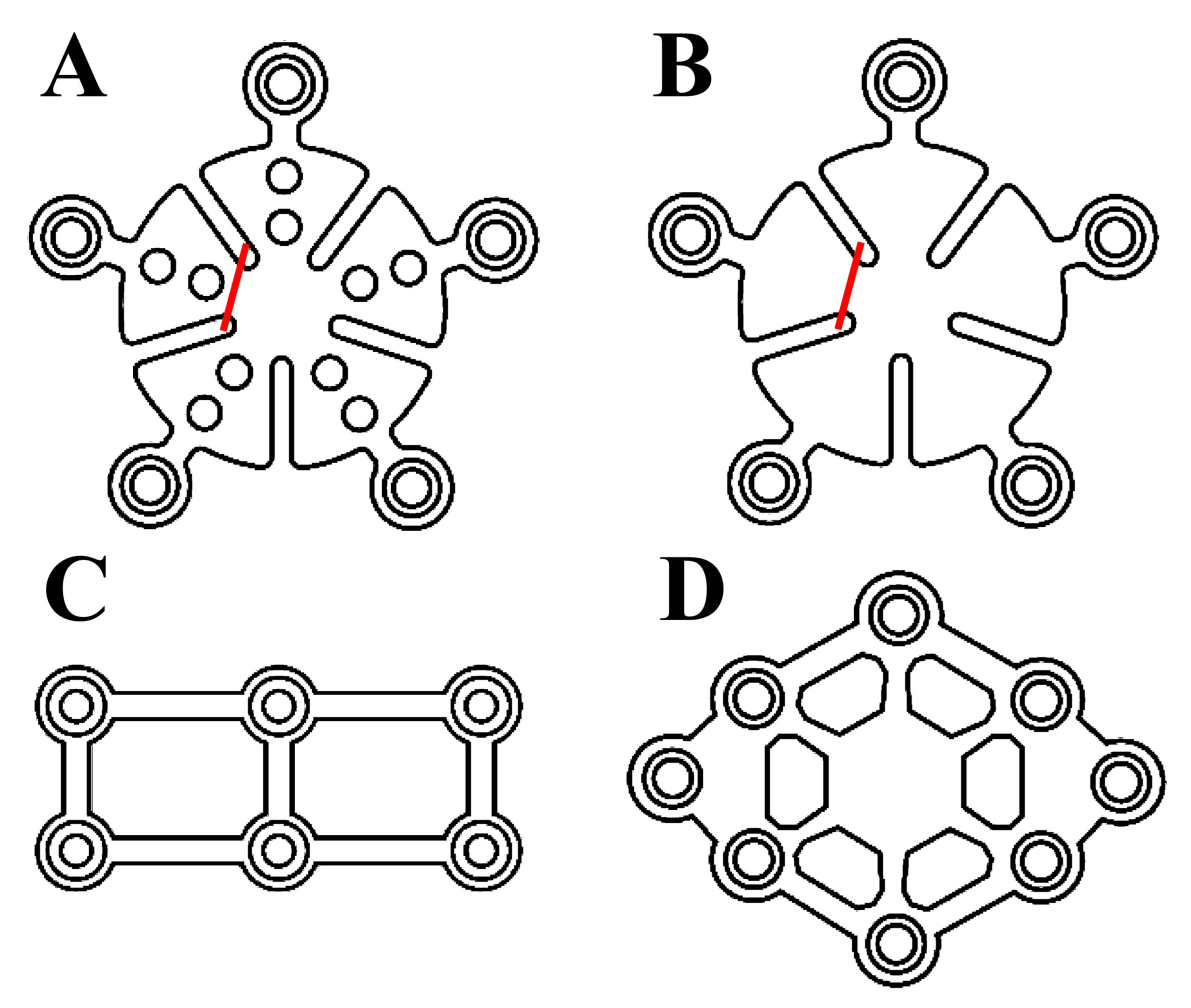
Titanium mini-plates of different shapes Image Credits: Yamada SM A, B: A pinwheel-shaped titanium mini-plate has five wings and five screw holes. If this pinwheel-shaped titanium mini-plate is used, it is better to fix all five screw holes for preventing lift-up when subjected to physical external forces. Alternatively, it is recommended to cut off the wing that is not screw-fixed at the point indicated by the red lines so that there are no bending parts. C, D: Using rectangular and diamond-shaped plates in the burr-hole area is also a good option. These mini-plates are affixed firmly to the skull/bone flap by fixing holes at the four corners with screws, without possessing parts that can be bent by external forces.

Failure to cover a burr hole when fixation of a bone flap in craniotomy results in scalp depression and skin thinning over the area, which is cosmetically problematic; therefore, titanium plates are routinely placed over these holes [7]. Titanium mini-plates, 0.5 mm in thickness, used to be standard; however, mini-plates with a thickness of 0.3 mm are now more commonly used for cranial fixation and plastic surgery [5,11]. Thinner plates have several advantages: they fit the skull shape easier, are less noticeable through the scalp, and the fixation force is not so reduced. However, a thinner plate itself is inevitably weaker [8]. When using a five-screw hole pinwheel-shaped titanium mini-plate to cover the burr hole (Figures 3A, 3B), it is common to use only three or four screw holes to fix the plate tightly to the skull/bone flap [10]. However, as illustrated by our patient and a similar previously reported case [10], titanium mini-plate scalp protrusion may occur after head trauma when all five screw holes are not used for fixation. Fortunately, our patient healed completely after plate removal. However, had an infection developed, bone flap removal and subsequent cranioplasty may have been required.

In general, when three of the five screw holes are fixed, the thin titanium mini-plate is so close to the skull that it cannot be inverted without an extremely thin detacher being inserted under the plate and lifted, and it seems improbable that it could be easily bent by a minor head injury. The authors consider that it is difficult to explain plate protrusion only by accidentally hitting the area that deforms the plate. It might be inevitable that the craniotomy-performed scalp will be more ischemic than a healthy scalp, as it is detached from the skull once and subsequently covers the bony fragment, and prolonged ischemia is likely to result in a thinner scalp than a healthy one. It cannot be ruled out that the long-term screw fixation of the mini-plate may have created a small gap between the plate and the skull. Our case occurred 17 years after craniotomy, and a previously reported case had a minor head injury 16 years after craniotomy [10]. Furthermore, our case had been undergoing urinary dialysis for more than 10 years, which may be related to the thinning and fragility of his scalp. However, if all five screw holes had been used for fixation, the likelihood of the plate and bone shifting over time would have been reduced and the risk of the plate bending due to a head injury would have been very low.

When performing craniotomy with subsequent bone flap fixation, neurosurgeons need to provide measures so that the titanium plate will not bend by minor head trauma. Gupta et al. stopped placing a full-size burr hole in the keyhole when performing frontotemporal craniotomy, eliminating the need for a plate [1]. When a burr-hole cover is required, all screw holes should be secured with screws [10]. Alternatively, if a pinwheel-shaped plate is being used, we suggest cutting the wing that is not fixed with a screw (Figures 3A, 3B). A rectangular or diamond-shaped plate (Figures 3C, 3D) with a bend-resistant shape could be used as well. Bone flap fixation is a procedure that is performed after the tension in the microscope manipulation has been released and can be performed with relaxation. However, bone flap fixation requires careful attention for an acceptable outcome. The current case suggests further research areas such as testing the mechanical properties of different mini-plate designs and conducting retrospective studies of long-term plate-related complications.

## Conclusions

Titanium mini-plates are essential to cover burr holes in craniotomy, and pinwheel-shaped titanium plates of 0.3-mm thickness are frequently utilized considering cosmetic aspects. However, the plates themselves are not strong, possessing a risk of bending when subjected to an external force. Head contusion that causes the plate to bend is extremely rare, but great care should be taken for bone flap fixation. Pinwheel-shaped burr-hole plates should be fixed using all screw holes or the wing that is not fixed should be cut. Alternatively, a rectangular or diamond-shaped plate should be used.
